# Does the conventional landmark help to place the tip of REBOA catheter in the optimal position? A non-controlled comparison study

**DOI:** 10.1186/s13017-019-0255-0

**Published:** 2019-07-16

**Authors:** Kento Nakajima, Hayato Taniguchi, Takeru Abe, Keishi Yamaguchi, Tomoki Doi, Ichiro Takeuchi, Naoto Morimura

**Affiliations:** 1 Advanced Critical Care and Emergency Center, Yokohama City University Medical Center, 4-57 Urafunecho, Minami-ku, Yokohama, Kanagawa 232-0024 Japan; 2 Department of Emergency Medicine, Graduate School of Medicine, Yokohama City University, 3-9 Fukuura, Kanazawa-ku, Yokohama, Kanagawa 236-0004 Japan; 3 Department of Surgery Intensive Care, Nippon Medical School Hospital, 1-1-5 Sendagi, Bunkyo-ku, Tokyo, 113-8603 Japan; 4 Critical Care and Emergency Center, Yokosuka Kyosai Hospital, Yonegahama Street 1-16, Yokosuka, Kanagawa 238-8558 Japan; 5 Department of Acute Medicine, Graduate School of Medicine, The University of Tokyo, 7-3-1 Hongo, Bunkyo-ku, Tokyo, 113-8655 Japan

**Keywords:** Torso trauma, Hemorrhagic shock, Resuscitative endovascular balloon occlusion of the aorta

## Abstract

**Background:**

Resuscitative endovascular balloon occlusion of the aorta (REBOA) for patients with traumatic torso hemorrhagic shock is available to keep a minimum level of circulatory status as a bridge to definitive therapy. However, the trajectory for placement of REBOA in the aorta has not yet been clearly defined.

**Methods:**

We conducted a retrospective observational cohort study in the two tertiary critical care and emergency center from December 2014 to October 2018. A total of 28 patients who underwent focused assessment with sonography for trauma (FAST) were studied via contrast computed tomography (CT), and 27 were analyzed.

**Results:**

We divided patients into two groups based on our CT findings. The REBOA deflate group included 16 patients, and the inflate group included 11 patients. The median trace value (interquartile range) of the blood vessel center line from the common femoral artery to the tip of REBOA (blood vessel length) and the length of REBOA itself from the common femoral artery to the tip of REBOA (REBOA insertion length) were 56.2 cm (54.5–57.2) and 55.2 cm (54.2–55.6), respectively (*p* < 0.0001) for the deflated group, and 51.4 cm (42.1–56.6) and 50.3 cm (42.3–55.0) (*p* = 0.594), respectively, for the inflated group.

**Conclusions:**

If REBOA was deflated, it was placed 1.0 cm longer than the insertion length of REBOA catheter itself, but that was not the case when inflating REBOA. The individual difference was large to the extent that the balloon inflated and the extent to which the balloon was pushed back toward the caudal depending on the degree of blood pressure. Further studies would be needed to validate the study findings.

## Background

Hemorrhagic shock is a major cause of traumatic death [1–7]. To avoid trauma death, it is important to stop bleeding as soon as possible. Resuscitative endovascular balloon occlusion of the aorta (REBOA) for patients with traumatic torso hemorrhagic shock is available to keep a minimum level of circulatory status as a bridge to definitive therapy [2, 8–12].

Especially for profound shock patients, the prompt placement of REBOA is essentially important. However, in such situations, there are limitations of time and equipment to accurately place REBOA, which is ideally placed under fluoroscopy. To date, using the mid-sternum as a landmark, and inserting REBOA, the length from the thigh to the mid-sternum is implied in the range of aortic zone I [13]. If the insertion length of REBOA is longer than the length from the thigh to the xiphoid process in the body surface and is shorter than the length from the thigh to the sternum notch, the tip is placed in the aortic zone I [14]. In addition, morphometric roadmaps have been identified to keep REBOA in an exact zone under non-fluoroscopy [15].

However, the trajectory of where to place REBOA in the aorta has not been clearly defined [16]. For example, it is expected that the position of the tip is different from the estimated value on the desk due to inflation or deflation of the balloon, circulation dynamics, and the like. There is no margin of time to consider during the resuscitation of patients with severe trauma, and an indicator to predict how close to the target zone REBOA will reach under non-fluoroscopy is required. Thus, in this study, we identified how REBOA traveled through the aorta and where the tip was located, and how much it deviated from the estimated value in the reconstructed computed tomography (CT). Our study hypothesis was that blindly but safely placed REBOA in the targeted zone can be inserted at different distances depending on whether REBOA is inflated or deflated. Our findings can enable practitioners to obtain a more precise REBOA placement distance, consequently leading to a safer approach that is unaffected by institutional or personnel variability.

## Methods

### Patients and study setting

This was a retrospective observational cohort study. We targeted trauma patients who underwent focused assessment with sonography for trauma (FAST) and transported to Yokohama City University Medical Center’s Advanced Critical Care and Emergency Center, in Yokohama City (YCU), and Yokosuka Kyosai Hospital Critical Care and Emergency Center, in Yokosuka City (YKH), Japan, from December 2014 to October 2018. The study was approved by the institutional review boards at both institutes.

The population of Yokohama City was 3,740,944 in 2019 [17], and there are nine critical care and emergency centers in the city. The population covered per emergency medical center would be approximately 415,660. Yokosuka City has two emergency medical centers, and those centers would be responsible for critical patients in Yokosuka City, adjacent Miura City, and Zushi City. The population of Yokosuka, Zushi, and Miura City was 497,452 from the latest data [18–20]. Thus, the population covered per emergency medical center would be approximately 248,726.

In the study institutions, the availability and immediacy of trauma surgeons and interventional radiology (IVR) physicians could vary the time until radical hemostasis. In addition, the length of time until a portable X-ray can be utilized and CT imaging can be conducted for each patient might also differ. In cases of shock due to severe trauma, without critical positive findings in head or chest trauma and with suspected bleeding in the abdominal or retroperitoneal cavity, REBOA can be placed in aortic zone I. In such cases, deflated REBOA could be used as a bridge to definitive hemostasis. In cases of negative FAST and intra-abdominal hemorrhage, REBOA can be placed in aortic zone III. However, to rapidly place REBOA under non-fluoroscopy, it can initially be positioned in the wider aortic zone I. After CT imaging and diagnosis, the placement position can be changed to aortic zone III depending on treatment necessity.

**Fig. 1:**
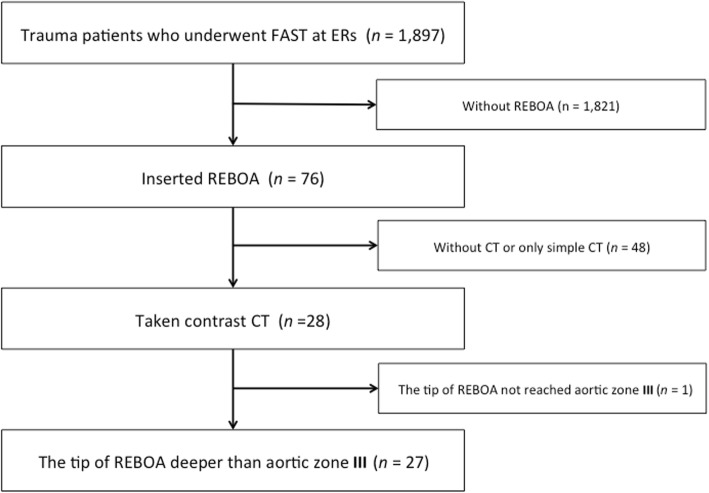
Flow chart of patient inclusion in this study. Of the 1897 patients in two facilities, and among the 76 patients in whom REBOA was inserted, a total of 28 patients had taken a contrast CT. The inserted length of REBOA itself and the center line of the blood vessel from the common femoral artery to the tip of REBOA would be equivalent to the blood vessel length. We excluded one patient whose tip of REBOA did not reach aortic zone III and analyzed 27 patients

**Fig. 2:**
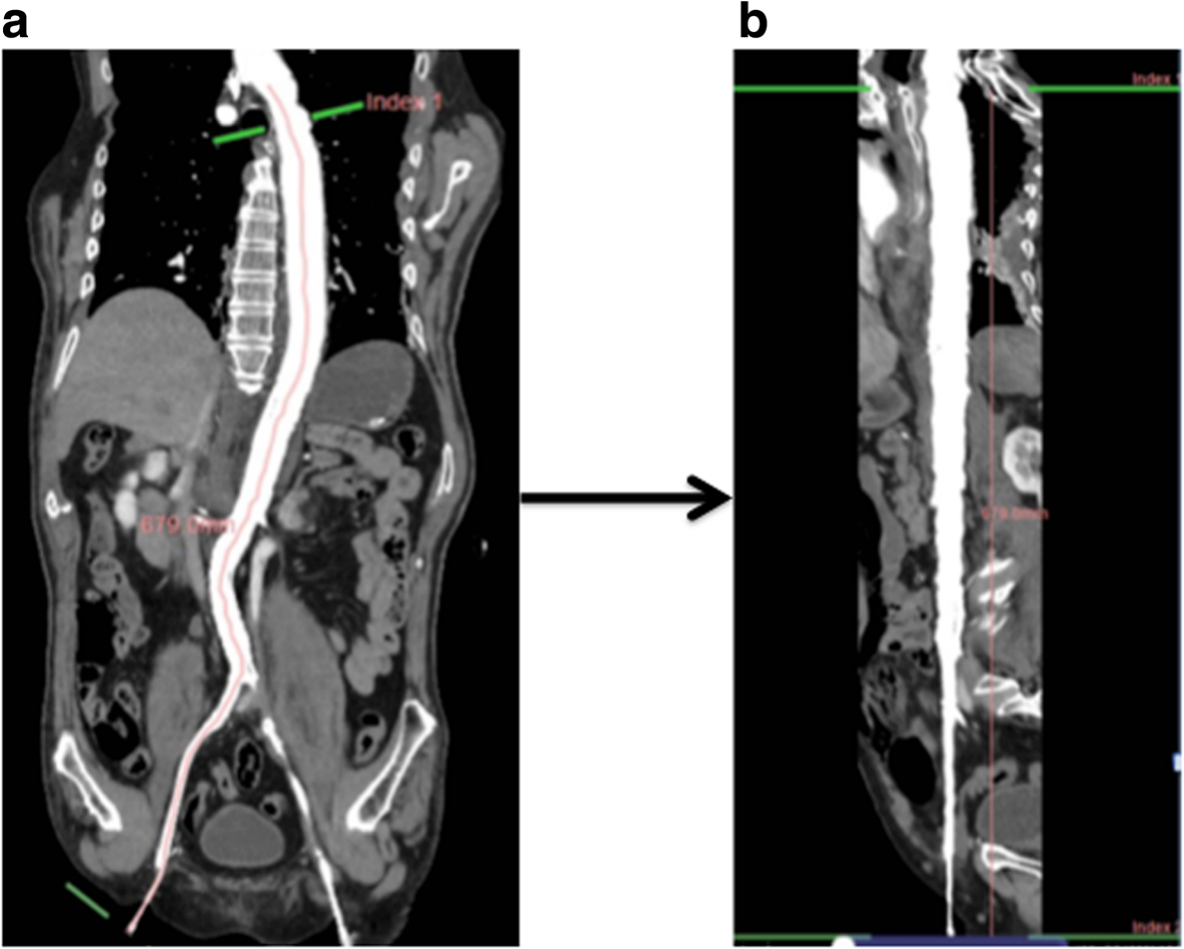
How to measure the blood vessel length using Ziostation 2 PLUS. **a** We plotted the center point of the contrasted vascular lumen from the common femoral artery to the blood vessel cross section at the tip of REBOA at the horizontal disconnection of CT. **b** We reconstructed meandering blood vessels approximately linearly and measured the blood vessel length

The exclusion criteria were patients under 15 years of age, without FAST enforcement, without REBOA insertion, without CT imaging, or only simple CT imaging. The patients with REBOA which did not reach aortic zone III were excluded. Of the 1897 patients in two facilities, and among the 76 patients in whom REBOA was inserted, a total of 28 patients had taken a contrast CT. The inserted length of REBOA itself and the center line of the blood vessel from the common femoral artery to the tip of REBOA would be equivalent to the blood vessel length. We excluded one patient whose tip of REBOA did not reach aortic zone III in three-dimensional (3D) medical images, and analyzed 27 patients (Fig. 1). Measurement was performed using data processing software (Ziostation 2 PLUS, Ziosoft Corp., Tokyo, Japan). We plotted the center point of the contrasted vascular lumen from the common femoral artery to the blood vessel cross section at the tip of REBOA at the horizontal disconnection of CT. Then, we reconstructed meandering blood vessels approximately linearly and measured the blood vessel length (Fig. 2). In this study, systolic blood pressure of 90 mmHg or less was defined as low blood pressure [2, 21].

### Indication and procedure

**Fig. 3:**
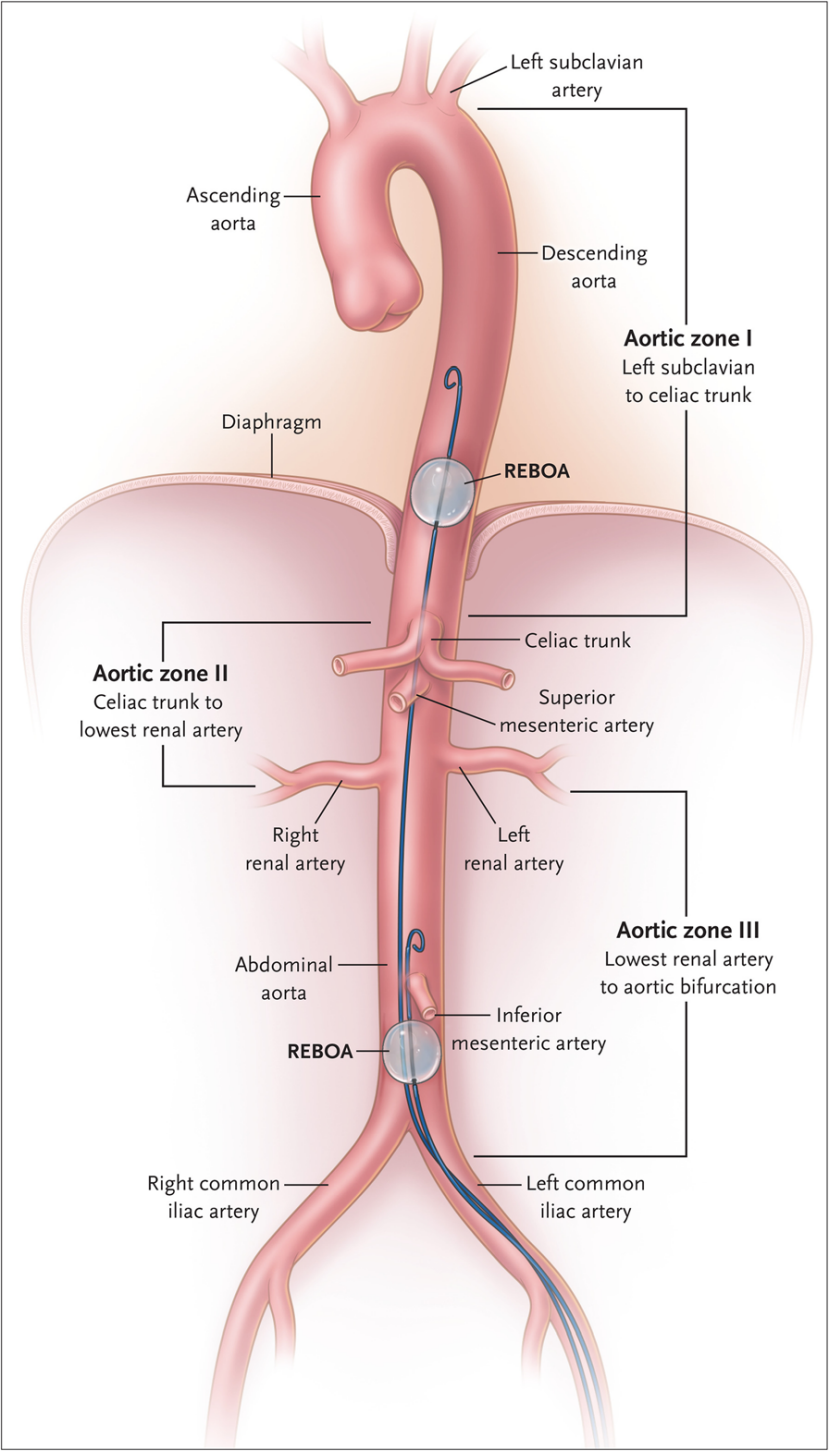
Classification of aortic zone. Aortic zone I extends from the origin of the left subclavian artery to the celiac artery. Aortic zone II extends from the celiac artery to the lowest renal artery. Aortic zone III exists from the lowest renal artery to the aortic bifurcation. From King DR. Initial care of the severely injured patient. N Engl J Med 2019; 380(8):763-70. Copyright © 2019 Massachusetts Medical Society. Reprinted with permission from Massachusetts Medical Society

The aorta is classified into three zones for the purpose of REBOA insertion. Aortic zone I extends from the origin of the left subclavian artery to the celiac artery. Aortic zone II extends from the celiac artery to the lowest renal artery. Aortic zone III exists from the lowest renal artery to the aortic bifurcation (Fig. 3) [12, 22]. We also defined a zone which exceeded aortic zone I as aortic zone 0. REBOA is mainly placed in aortic zone I for intra-abdominal bleeding control and in aortic zone III for pelvic fracture and control of lower limb bleeding. However, in emergency situations, REBOA is often placed in aortic zone I for the time being [2, 23–25].

In patients requiring REBOA, we first inserted a 4- to 6-Fr short percutaneous sheath from the common femoral artery and exchanged it to a 7-Fr short sheath [26]. The femoral artery is the most common access site [21]. Next, the guide wire was advanced, and REBOA was placed blindly under non-fluoroscopy by the emergency physician. We used a small-diameter 7-Fr Rescue Balloon™ or Rescue Balloon ER™ (Tokai Medical Products Corp., Kasugai, Aichi, Japan).

### Data collection

Patient characteristics (age, sex, height, and racial group), mechanism of injury, patient vital signs, FAST whether positive or negative, injury severity score (ISS) [27], left or right approach to the common femoral artery, the aortic zone in which the REBOA tip was located, whether REBOA was inflated or deflated, and the outcome were collected from the medical records. The ISS was calculated for each patient.

### Statistical analysis

We obtained descriptive statistics, such as median and interquartile range for continuous variables, and frequency and proportion for categorical variables, and compared them between the inflate and deflate groups. We used the Mann-Whitney *U* test for continuous variables, and the Fisher exact test for categorical variables. The clinical characteristics of all included cases were described. The trace value (vascular length) by the blood vessel center line from the common femoral artery to the tip of REBOA and the length of REBOA itself (REBOA insertion length) from the common femoral artery to the tip of REBOA were divided into the inflate group and the deflate group and examined using the related-samples Wilcoxon signed-rank test. A compatibility test was conducted for statistical analysis, and two-sided *p* < 0.05 was regarded as significant. All analyses were performed using IBM SPSS ver. 23 (IBM Corp., Armonk, NY, USA).

## Results

The median (interquartile range) age of patients was 43 years (34–59.5), and 23 patients (85.2%) were male. There were 26 blunt traumas (96.3%). The mechanism of the injury consisted of 10 falls (37.0%), 7 traffic injury (25.9%), 5 pedestrian injury (18.5%), 3 train injury (11.1%), 1 compression (3.7%), and 1 gunshot wound (3.7%). The mean ISS was 31.4. Among all patients, 10 patients (37.0%) died in the hospital (Table 1).

**Table 1:** Characteristics of patients who were inserted with REBOA and have taken contrast CT

	Inflate (*n* = 11)	Deflate (*n* = 16)
Age (years)	43 (29.5–59.5)	43 (37–57.25)
Male, *n* (%)	10 (90.9%)	13 (81.3%)
Blunt injury, *n* (%)	11 (100%)	15 (93.8%)
Mechanism of injury, *n* (%)		
Fall	3 (27.3%)	7 (43.8%)
Traffic	1 (9.1%)	6 (37.5%)
Pedestrian	4 (36.4%)	1 (6.25%)
Train	3 (27.3%)	0 (0%)
Compression	0 (0%)	1 (6.25%)
Gunshot	0 (0%)	1 (6.25%)
ISS	26 (20.5–37.5)	29 (26–35)
In-hospital mortality, *n* (%)	5 (45.5%)	5 (31.3%)
Blood vessel length (cm)	51.4 (42.1–56.6)	56.2 (54.5–57.2)
REBOA insertion length (cm)	50.3 (42.3–55.0)	55.2 (54.2–55.6)

For age, ISS, blood vessel length, and REBOA insertion length, medians and interquartile ranges (25th–75th percentile) are shown

Table 2 shows the characteristics of 27 patients. The mean values for systolic blood pressure, heart rate, and respiratory rate were 65.8 mmHg, 101.2 bpm, and 23.7/min, respectively. There were 26 patients on REBOA that was placed in aortic zone I, and one had been placed on the head side of the left subclavian artery bifurcation. The insertion site of REBOA was from the right common femoral artery in 12 patients and from the left common femoral artery in 15 patients. The REBOA deflate group included 16 patients; the inflate group included 11 patients.

**Table 2:** Patients included in this study

No.*	Age	Sex	Height (cm)	Mechanism of injury	ISS	Aortic zone**	Femoral approach	Blood vessel length (cm)	REBOA insertion length (cm)	Inflate or deflate	FAST
1	70	M	170	Fall	29	I	Left	57.0	55.4	Deflate	Positive
2	56	M	–	Compression	26	I	Left	57.2	55.7	Deflate	Positive
3	18	M	171	Traffic	22	I	Left	57.5	55.1	Deflate	Negative
4	45	M	–	Pedestrian	50	I	Left	56.2	55.9	Deflate	Negative
5	34	M	172	Gunshot	22	I	Left	55.8	55.1	Deflate	Positive
6	61	M	182	Traffic	26	I	Left	56.3	55.1	Deflate	Positive
7	32	M	–	Pedestrian	50	I	Right	55.3	52.9	Inflate	Positive
8	42	M	–	Fall	34	I	Left	57.8	57.4	Deflate	Positive
9	69	M	163	Traffic	17	I	Left	56.7	56.1	Inflate	Negative
10	38	M	–	Fall	29	I	Left	56.3	55.0	Deflate	Negative
11	43	M	166	Fall	26	I	Right	56.2	55.5	Deflate	Positive
12	43	M	–	Traffic	41	I	Right	49.9	49.6	Deflate	Positive
13	39	F	–	Fall	41	I	Right	55.7	55.3	Deflate	Positive
14	34	M	–	Traffic	34	I	Right	54.2	54.0	Deflate	Positive
15	58	F	–	Fall	66	I	Left	52.4	50.3	Inflate	Negative
16	61	M	172	Pedestrian	26	I	Right	56.6	55.0	Inflate	Negative
17	43	M	–	Train	26	I	Left	42.1	42.3	Inflate	Negative
18	69	M	173	Traffic	35	I	Right	56.8	55.4	Deflate	Negative
19	27	M	173	Fall	24	I	Left	64.3	63.9	Inflate	Negative
20	61	M	168	Pedestrian	33	I	Right	39.0	39.5	Inflate	Positive
21	49	M	175	Train	10	I	Left	43.4	43.8	Inflate	Negative
22	50	M	171	Fall	35	0	Right	58.8	58.7	Deflate	Negative
23	19	M	–	Traffic	41	I	Left	47.5	47.8	Inflate	Positive
24	88	F	148	Fall	27	I	Right	45.2	43.5	Deflate	Negative
25	20	M	–	Fall	16	I	Right	40.5	41.4	Inflate	Negative
26	42	M	–	Train	34	I	Right	51.4	52.0	Inflate	Negative
27	18	F	–	Traffic	27	I	Left	42.4	42.1	Deflate	Positive

*FAST* focused assessment with sonography for trauma

*Case 6 was Caucasian, and all of the others were Asian

**This study defined aortic zone 0 as placement on the head side of the left subclavian artery bifurcation, which is a non-standard location. Case 22 was not inflated, and no complications occurred

A total of 16 patients had deflated REBOA during CT imaging, and 13 patients (81.3%) had low blood pressure. Six patients (37.5%) had a head abbreviated injury score (AIS) greater than or equal to 3. The mortality rate was 31.3% (five patients), including two patients (12.5%) who were in cardiopulmonary arrest on arrival. The mean ISS was 31.5. There were 15 patients who had REBOA placed in aortic zone I, and the remaining 1 patient was placed on the head side from the branch of the left subclavian artery. The median trace value (interquartile range) of the blood vessel center line from the common femoral artery to the tip of REBOA (blood vessel length) and the length of REBOA itself from the common femoral artery to the tip of REBOA (REBOA insertion length) were 56.2 cm (54.5–57.2 cm) and 55.2 cm (54.2–55.6 cm), respectively (*p* < 0.0001).

A total of 11 patients had inflated REBOA during CT imaging. Ten patients (90.9%) had low blood pressure. In three patients (27.3%), the head AIS was larger than or equal to 3. The mortality rate was 45.5% (five patients), including two patients (18.2%) who were in cardiopulmonary arrest on arrival. The mean ISS was 31.2. REBOA were all placed in aortic zone I. The median trace value (interquartile range) of the blood vessel center line from the common femoral artery to the tip of REBOA (blood vessel length) and the length of REBOA itself from the common femoral artery to the tip of REBOA (REBOA insertion length) were 51.4 cm (42.1–56.6 cm) and 50.3 cm (42.3–55.0 cm) (*p* = 0.594), respectively.

## Discussion

In our study, we reconstructed the CT data and confirmed the trajectory of REBOA in the blood vessel. We first found that compared to the center line of the blood vessel, REBOA traveled more linearly, and if the balloon was deflated when REBOA was inserted, it was placed 1.0 cm longer than the insertion length of REBOA. On the other hand, that was not the case when inflating the balloon. This seems to suggest that REBOA travels more linearly in meandering blood vessels. Our findings can enable practitioners to obtain a more precise REBOA placement distance, consequently leading to a safer approach that is unaffected by institutional or personnel variability.

From the results of this study, we found that it is important to always assume the possibility of placement distal to the blood vessel, rather than the assumed insertion length when inserting REBOA under non-fluoroscopy. In this study, no fatal complications accompanying placing REBOA were observed. In addition, one case in which REBOA had been detained beyond aortic zone I was also included.

A previous study established a method of using landmarks of the body surface under non-fluoroscopy when inserting REBOA. If the insertion length was longer than the length from the thigh to the xiphoid process and it is shorter than the length from the thigh to the sternum notch, REBOA was placed in aortic zone I [15]. Another study using cadavers established a method using landmarks on the body surface, in which REBOA is placed in aortic zone I when inserted from the length of the thigh to the mid-sternum [14]. The method of using landmarks on the body surface seems to be a simple and easy-to-use method that does not require special devices and knowledge. There is a possibility that it can be applied in emergency outpatient or prehospital situations in which fluoroscopy or simple X-ray are not available [28]. However, it is difficult to estimate where the actual tip of REBOA is located, because the influence of the hemodynamics and degree of balloon dilation is not taken into consideration [16]. Therefore, to understand the dynamics of REBOA in a blood vessel, this study confirmed the position of its tip in cases in which REBOA had been inserted. In the deflated group of REBOA, the median (interquartile range) of the trace value (blood vessel length) by the blood vessel center line from the common femoral artery to the tip of REBOA and the length of REBOA itself from the common femoral artery to the tip of REBOA of the insertion length were 56.2 cm (54.5–57.2 cm) and 55.2 cm (54.2–55.6 cm) (*p* < 0.0001), respectively, and as a median, the difference was 1.0 cm. This means that the tip is significantly deeper by 1.0 cm as a median than the assumed length, when REBOA is inserted from the common femoral artery. The possible explanation would be that the running of REBOA and the center line of the aorta would not coincide with each other. REBOA would travel in the blood vessel more linearly by entering the metallic stylet inside the catheter. In addition, there was a tendency to travel as if it touched the aorta wall in a shortcut, and as a result, it was placed at a position deeper than the assumed REBOA insertion length. In the inflated group of REBOA, the median (interquartile range) of the trace value (blood vessel length) by the blood vessel center line from the common femoral artery to the tip of REBOA and the length of REBOA itself from the common femoral artery to the tip of REBOA of the insertion length were 51.4 cm (42.1–56.6 cm) and 50.3 cm (42.3–55.0 cm), respectively, and there was no significant association (*p* = 0.594). The possible explanation would be that the individual difference was large to the extent that the balloon inflated and the extent to which the balloon was pushed back toward the caudal depending on the degree of blood pressure.

There are several limitations in this study to mention. First, the length of the aorta and the degree of meandering might vary depending on age, sex, race, and other factors. Second, we did not investigate how far the balloon was inflated in the REBOA inflated group. Third, the current treatment procedure may not be common at other facilities and overseas, which will affect the external validity of this study’s findings. However, in several Japanese tertiary care emergency centers, REBOA was used by emergency physicians without fluoroscopic guidance and placed in a safe location with subsequent confirmation via CT [23]. Fourth, in this procedure, a wire is left at the time of measurement. There is a possibility that REBOA might be pushed distally by the flow, which could not be controlled in the current study design. Thus, further research is necessary to evaluate the flow mechanism. Fifth, since all of the cases but one were Asian, there were limitations to the external validity. Thus, the study findings might not apply to other racial groups. Finally, future international studies with larger sample sizes and more facilities are desirable to improve the representability and the generalizability of the findings.

## Conclusions

If REBOA was deflated, it was placed 1.0 cm longer than the insertion length of REBOA itself, but that was not the case when inflating REBOA. The individual difference was large to the extent that the balloon inflated and the extent to which the balloon was pushed back toward the caudal depending on the degree of blood pressure. Further studies are needed to validate our findings.

## Data Availability

The datasets used during the current study are available from the corresponding author on reasonable request.

## Abbreviations

3D: Three-dimensional
AIS: Abbreviated injury score
CT: Computed tomography
FAST: Focused assessment with sonography for trauma
ISS: Injury severity score
IVR: Interventional radiology
REBOA: Resuscitative endovascular balloon occlusion of the aorta
YCU: Yokohama City University
YKH: Yokosuka Kyosai Hospital
